# Influence of Childhood Adversity and Infection on Timing of Menarche in a Multiethnic Sample of Women

**DOI:** 10.3390/ijerph18084080

**Published:** 2021-04-13

**Authors:** Ayana K. April-Sanders, Parisa Tehranifar, Erica Lee Argov, Shakira F. Suglia, Carmen B. Rodriguez, Jasmine A. McDonald

**Affiliations:** 1 Division of Cardiovascular Medicine, Albert Einstein College of Medicine, Bronx, NY 10461, USA; ayana.aprilsanders@einsteinmed.org; 2 Department of Epidemiology, Mailman School of Public Health, Columbia University Irving Medical Center, New York, NY 10032, USA; pt140@columbia.edu (P.T.); ejl2152@cumc.columbia.edu (E.L.A.); shakira.suglia@emory.edu (S.F.S.); cbr2121@cumc.columbia.edu (C.B.R.); 3 Herbert Irving Comprehensive Cancer Center, Columbia University Irving Medical Center, New York, NY 10032, USA; 4 Department of Epidemiology, Rollins School of Public Health, Emory University, Atlanta, GA 30322, USA

**Keywords:** menarche, racial and ethnic minority, immigrant, early life

## Abstract

Childhood adversities (CAs) and infections may affect the timing of reproductive development. We examined the associations of indicators of CAs and exposure to tonsillitis and infectious mononucleosis (mono) with age at menarche. A multiethnic cohort of 400 women (ages 40–64 years) reported exposure to parental maltreatment and maladjustment during childhood and any diagnosis of tonsillitis and/or mono; infections primarily acquired in early life and adolescence, respectively. We used linear and relative risk regression models to examine the associations of indicators of CAs individually and cumulatively, and history of tonsillitis/mono with an average age at menarche and early onset of menarche (<12 years of age). In multivariable models, histories of mental illness in the household (RR = 1.44, 95% CI: 1.01–2.06), and tonsillitis diagnosis (RR = 1.67, 95% CI: 1.20–2.33) were associated with early menarche (<12 years), and with an earlier average age at menarche by 7.1 months (95% CI: −1.15, −0.02) and 8.8 months (95% CI: −1.26, −0.20), respectively. Other adversities indicators, cumulative adversities, and mono were not statistically associated with menarcheal timing. These findings provided some support for the growing evidence that early life experiences may influence the reproductive development in girls.

## 1. Introduction

Puberty is a pivotal period for human growth and development that culminates in sexual maturity [1]. In girls, this development is a continuous process and is typically characterized first by breast development, followed by pubic hair growth, and the initiation of the menstrual cycle (or menarche), with the latter being one of the final secondary sex characteristics of pubertal maturation [1]. Extensive evidence indicates that the age at pubertal timing (commonly measured as age at menarche) has been declining over the last century [2]; an occurrence that has been largely attributed to secular trends in childhood environmental factors and exposures [3,4,5]. In the U.S., the average age at menarche is approximately 12.7 years among non-Hispanic white girls, while non-Hispanic black and Hispanic/Latina girls are more likely to have menarche earlier (12 years and 11.8 years, respectively) and Asian girls have menarche slightly later (12.8 years) [6]. Early pubertal maturation in girls (<12 years of age) is a noteworthy public health concern because of its link to a range of adverse health outcomes throughout the life course, including internalizing and externalizing behavioral problems [7], early sexual activity [7], poor mental health [8] in adolescence, and with chronic diseases such as cardiovascular disease [9,10] and breast cancer [11] in adulthood.

The determinants of early age at pubertal maturation have been studied extensively in developed countries [12,13,14], and research in developing nations is recently emerging [15,16]. Early life experiences, such as exposures to socioeconomic and psychosocial adversity, have been proposed as possible factors influencing pubertal timing through activation of stress-response systems that may directly influence dysregulation of hormonal and developmental processes [3,17]. Childhood adversity refers to childhood experiences (<18 years old) that threaten the child’s bodily, familial, or social safety or security and are recognized as a crucial early life environmental component influencing a range of adverse outcomes across the life course [18,19,20,21]. Factors like childhood abuse and other forms of household dysfunction are associated with poor physical health outcomes in childhood and adolescence [22], as well as through adulthood [23]. Individual indicators of childhood adversity were linked with the timing of pubertal development, but the findings were inconsistent. A recent systematic review and meta-analysis of adverse childhood experience (ACEs) and early pubertal timing in girls found that sexual abuse, father’s absence from the household, and family dysfunction were significantly associated with early pubertal timing among girls; cumulative ACEs was not associated with early pubertal timing [24]. However, a recent study of the Puerto Rican youth found that cumulative childhood adversities were associated with earlier pubertal maturation in girls [25], which may reflect differences in the type of measures used to assess pubertal development. In the review and meta-analysis study by Zhang et al. [24], the authors observed great heterogeneity between estimates for all the outcomes measured, and this was largely due to variability in measurement of childhood adversities and puberty across studies, sociodemographic variability in study samples, and study design (cross-sectional or longitudinal). Therefore, findings of the relationship between childhood adversities and pubertal timing are still inconclusive and warrant continued assessment to help identify distinct dimensions, duration, and sensitive periods of childhood adversities that may differentially influence pubertal maturation in girls from diverse backgrounds [24].

In addition to childhood adversity, other childhood exposures changed trajectories over the last century and may also explain the current trends of declining age at menarche. A significant focus was placed on the relationship between childhood BMI and secular trends of pubertal timing. Findings showed that there was an association between adiposity and menarche bolstered by the fact that an increased BMI preceded early puberty in girls; therefore, suggesting that BMI may plausibly contribute to earlier menarche [26,27,28]. However, the trend of early puberty existed in populations with and without the childhood obesity epidemic [29]. In particular, the decline in age at menarche predated the childhood obesity epidemic by seven decades and, thus, suggests that factors other than childhood BMI may contribute to early menarche [3]. As such, studies did not establish a direct, causal relationship between childhood obesity and early puberty. A general decline in the prevalence and average age at acquisition of many childhood infections [3,30] may provide another explanation for changes in age at menarche. Two prevailing yet contradictory theories suggested either that reproduction was prioritized in a low childhood infections exposure environment [1,31], or that, alternatively, childhood viral exposures were obesogenic and were drivers in the association between increased obesity and earlier pubertal maturation [32,33,34]. In support of the former theory, both HIV [35] and Hepatitis B [36] infections during childhood were associated with later age at menarche, although empirical research remains limited.

In the present study, we focused on commonly contracted childhood viral infections: tonsillitis and infectious mononucleosis. In particular, Epstein–Barr Virus (EBV) exposure, which is typically acquired in early life, was of growing interest given the existing clinical trials assessing a prophylactic EBV vaccine [37] to potentially reduce the incidence of infectious mononucleosis. For instance, where early life and childhood EBV infections are persistent and mostly latent (e.g., dormant or inactive), delayed primary EBV infections were associated with infectious mononucleosis, which elevated the risk of Hodgkin’s lymphoma and multiple sclerosis [38,39]. Further, studies also suggested that primary infections, with an emphasis on delayed primary infections, were associated with an increased breast cancer risk [40,41,42]. As in previous reports [43,44,45], using surrogate measures for EBV infection timing (i.e., doctor-diagnosed tonsillitis as a proxy for a primary EBV infection in early childhood and doctor-diagnosed infectious mononucleosis as a proxy for a delayed primary EBV infection in adolescence/early adulthood) [39,46] may help to distinguish early vs. delayed EBV infection, as only doctor-diagnosed tonsillitis would be expected prior to menarche.

The literature suggests that there is still limited knowledge about the early life influences of pubertal timing in socially diverse samples. On average, children from Hispanic and non-Hispanic black backgrounds and/or from families with lower socioeconomic position (SEP) have a higher prevalence of exposure to childhood adversities and infectious agents than non-Hispanic white and/or higher SEP children [47,48,49,50,51]. These groups also experience comparatively earlier pubertal timing in adolescence and are at an increased risk of chronic diseases in adulthood [10]. Life-course models recognized the opportunity to prevent and control diseases at key stages of life and established theoretical frameworks, like the critical period or accumulating risk hypotheses that may relate these factors and integrate the evidence linking early life adversities and infection to pubertal timing [52]. This perspective presented an opportunity to synthesize the literature on the antecedents of pubertal timing in light of compelling evidence supporting the role of stress and stress systems in setting an individual’s development on an accelerated or delayed reproductive life course with numerous physical and psychological outcomes.

In the present study, we sought to clarify the potential role of early life exposure to adversity and infections with the average and early (<12 years) age at menarche in a socially diverse sample of adult women recruited from a screening mammography clinic in New York City. We tested the following hypotheses: (1) exposure to childhood adversity (specific domains and types, cumulative) is associated with earlier age at menarche; and (2) tonsillitis (childhood primary infection), but not mono (adolescent/early adulthood primary infection), is associated with later age at menarche.

## 2. Materials and Methods

### 2.1. Study Population

We used data from the New York Mammographic Density Study, a study of breast cancer screening and prevention in women aged 40 to 64 years, recruited during routine screening mammography visits at a community clinic in New York City [53,54]. Of the 1343 women, who were enrolled and completed a baseline questionnaire between 2012 and 2018, 524 were presented with a follow-up questionnaire module on early life exposures, including childhood adversity and infection, of whom 401 (76.5%) completed the data. We excluded data from one woman with missing data for age at menarche. No systematic differences in age at menarche and sociodemographic characteristics were observed between the included (N = 400) and excluded (N = 123) subjects (Table A1). The Columbia University Institutional Review Board approved this study; all women provided written informed consent.

### 2.2. Measures

Childhood adversities. Exposure to childhood adversities was measured using seven self-reported items that evaluated two major childhood adversity domains experienced before the age of 18 years: (a) parental maltreatment (neglect and physical and emotional abuse) and (b) parental maladjustment (intimate partner violence, substance use problems, incarceration, and mental illness in the household). Each of the seven items was part of the original factors assessed in the Adverse Childhood Experiences Study [23] that focused on adversities in the household when the participant was under the age of 18 years. The accumulation of risk conceptual model postulated that correlated and independent risks could build over time to cumulatively influence health outcomes later in life; in other words, with more adverse exposures, we would expect greater health risks [52,55]. Using this model, we hypothesized that an increasing number of adverse events during childhood would be associated with a higher likelihood of earlier age at menarche. We categorized the number of cumulative childhood adversities into four groups (0 adversity, 1 adversity, 2 adversities, ≥3 adversities) for analysis. Individual indicators of childhood adversities were also examined (endorsement categorized as yes/no). For analyses modeling the effect of cumulative indicators of adversity, we excluded women with incomplete responses to the seven indicators (N = 57, analytic sample size N = 343). For analyses modeling individual indicators of adversity, we excluded women from analyses with incomplete responses for the specific measures (N missing ranging from 12 to 27 women per measures; analytic sample sizes ranged from N = 373 to N = 388 per adversity).

Infections. EBV infection is commonly acquired in early life, with peak acquisition between infancy and young adulthood [46]. Early life infection is often latent and asymptomatic; however, delayed or late primary infection is often associated with symptomatic disease [40,44]. Therefore, we used two surrogate measures to assess primary EBV infection at two points: early childhood and adolescence/early adulthood. Women were asked “Has a doctor or a healthcare professional ever told you that you had tonsillitis? Infectious mononucleosis?” We also provided a description for each disease. Tonsillitis is an inflammation of the tonsils, with most cases due to a viral infection such as EBV [56]. A major target site for EBV is the tonsillar tissue [57]. Infectious mononucleosis, also known as mono, which is associated with deep kissing, is associated with a delayed primary EBV infection in adolescence and/or early adulthood. For this exploratory analysis, we assumed that tonsillitis is an indicator of a childhood primary infection and mono is an indicator of an adolescent or early adulthood primary infection. Therefore, without definitive temporal information, we considered a delayed primary EBV infection to occur at a later age for women who report mono compared to those who report tonsillitis. We assumed that being diagnosed with tonsillitis would be prior to menarche. Under these assumptions, we created a categorical variable comparing reports of tonsillitis only, mono only, both tonsillitis and mono (reflecting early EBV infection due to tonsillitis), and neither tonsillitis nor mono (referent group). We also created a dichotomous variable comparing tonsillitis only to never tonsillitis. Analyses modeling infections were restricted to women missing neither tonsillitis nor mono data (N = 352).

Timing of menarche. Age at menarche was ascertained retrospectively (half years). We examined the age at menarche both continuously (years) and categorically (<12 years vs. ≥12 years), with the categorical division made based on the median age at menarche in the U.S.

Covariates. Participants’ sociodemographic information included age at interview, race/ethnicity (Hispanic, non-Hispanic white, non-Hispanic black, non-Hispanic other), educational attainment (high school diploma or less, greater than high school diploma), language dominance (English, Spanish), and nativity status (U.S.-born, foreign-born).

### 2.3. Statistical Analysis

We assessed descriptive statistics for sociodemographic characteristics, the timing of menarche, the prevalence of childhood adversities, and infection history using counts (percentages) for categorical measures and means (standard deviations) for continuous measures. We conducted multivariable analyses to test the associations between childhood adversities (cumulative and individual indicators) and exposure to childhood infections (independent and joint histories of tonsillitis and mono), with age at menarche. All regression analyses accounted for confounding covariates that were selected as *a priori* covariates (age and race/ethnicity) or through statistical assessment (education and language dominance). Multivariable linear regression was used with continuous measures of age at menarche, while multivariable relative risk regression was used when modeling early age at menarche (<12 years vs. ≥12 years). As a sensitivity analysis, we assessed the association between childhood adversity with menarcheal age using an alternate grouping of childhood adversity where cumulative adversity was characterized by domains: childhood maltreatment (physical and emotional abuse) and parental maladjustment (intimate partner violence, substance use problems, incarceration, mental illness in the household). All statistical tests were two-sided and performed using SAS 9.4 (SAS Institute, Gary, NC, USA).

## 3. Results

The distribution of sociodemographic characteristics, childhood adversities, and early childhood infections of women by menarche status (early and average/late) are shown in Table 1. The majority of the sample identified as Hispanic (73.5%), were educated beyond the high school level (57.3%), were foreign-born (66.3%), and were predominantly Spanish-speakers (53.0%). Childhood adversities were prevalent among the sample, with one-third reporting 2 or more adversities and almost 20% having 3 or more childhood adversities. Experiencing emotional (29.3%) and physical abuse (24%), and household alcohol abuse (20.5%) were the most commonly reported childhood adversities (Table 1). Twenty-six percent of women reported ever being diagnosed with tonsillitis or mono—tonsillitis most prevalent (21.3%)—and less than 5% of women reported having had both infections. The mean age at menarche was 12.6 years (SD ± 1.9), with nearly one-third of women reporting an early age at menarche or onset of menarche at under 12 years of age (Table 1). Differences in the characteristics between women reporting early age at menarche and those reporting average or late age were observed. The average age of menarche was 10.4 years (SD = 0.9) among women with early menarche, compared with 13.6 years (SD = 1.4) in women with average or late age at menarche. Tonsillitis was also more prevalent among the early menarche group than the average/late menarche group (24.2% vs. 13.0%), while the average/late menarche group was more likely to report having had mono than the early menarche group (5.4% vs. 3.2%).

In Table 2, we report age, race/ethnicity, education, and language dominance adjusted mean age at menarche predicted by childhood adversities from linear regression models. The average age at menarche did not differ by levels of cumulative childhood adversity (1, 2, ≥3) compared with no adversity. When observing the relationship between individual indicators of adversities and mean age at menarche, women reporting living with a parent/caregiver who was mentally ill or depressed in the household, compared with those not reporting this adversity, had approximately 7.1 months earlier age at menarche (95% CI: −1.15, −0.02). The average age at menarche did not differ for other indicators of childhood adversities. In Table 3, we report the adjusted mean age at menarche predicted by childhood infections from linear regression models. Women reporting ever being diagnosed with tonsillitis, compared with never being diagnosed with tonsillitis, had 8.8 months earlier age at menarche (95% CI: −1.26, −0.20). Similarly, exposure to tonsillitis, only compared with being diagnosed with neither tonsillitis nor mono, was associated with 6.7 months earlier age at menarche (b = −0.58, 95% CI: −1.07, −0.09). Mono was not associated with the mean age at menarche.

**Figure 1:**
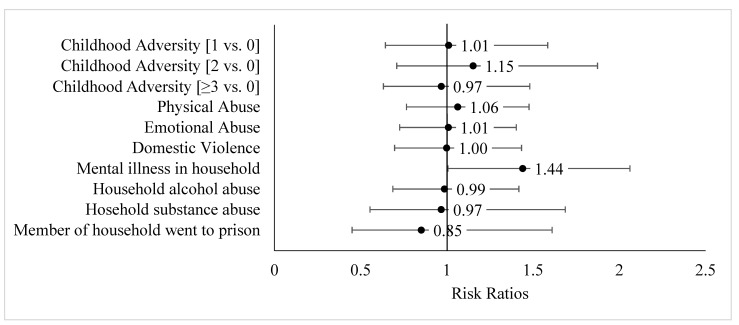
Adjusted associations of childhood adversity, cumulative and individual indicators, with early age at menarche (<12 years), relative risk ratios, and 95% confidence intervals. Notes: Models for cumulative childhood adversity indicators included complete responses for each of the seven adversity questions (N= 343). Models for individual childhood adversities included complete responses per indicator (range: N = 373 to 388); reference endorsed vs. not endorsed. All models adjusted for age at interview, race/ethnicity, education, and language dominance.

**Figure 2:**
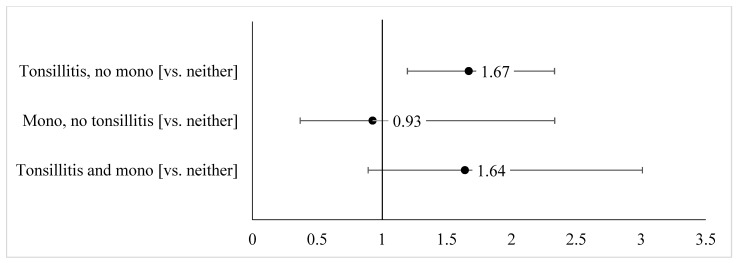
Adjusted associations of childhood infection (tonsillitis and mono) with early age at menarche (<12 years), relative risk ratios, and 95% confidence intervals. Notes: Models for childhood infection included complete responses for each of the infection history questions (N = 352). All models adjusted for age at interview, race/ethnicity, education, and language dominance. Model for ever vs. never tonsillitis also adjusted for ever mono. Model for ever vs. never mono also adjusted for ever tonsillitis.

In the multivariable relative risk regression models assessing the association between childhood adversity and early age at menarche (<12 years; Figure 1), we observed no significant associations between cumulative adversities (1, 2, ≥3 vs. 0 adversities) and early age at menarche. Only past exposure to mental illness in the household was associated with an increased likelihood of early age at menarche (RR = 1.44, 95% CI: 1.01, 2.06). In Figure 2, we estimated the association between childhood infection and early age at menarche in adjusted multivariable models. No association was observed between mono and early age at menarche. Having a history of being diagnosed with tonsillitis and no history of mono was associated with a 67% greater likelihood of early age at menarche compared with women with neither infection (95% CI: 1.20–2.33). 

In our sensitivity analysis, when grouping childhood adversities into domains of childhood maltreatment and parental maladjustment (N = 343), associations between childhood adversities and age at menarche were consistent with findings from the main analysis (Table A2 and Table A3).

## 4. Discussion

In this socially diverse sample of midlife women, we examined indicators of childhood adversities and infections and found moderate evidence that early life factors may affect the timing of pubertal development. Women experiencing an early age at menarche were more likely to have lived in a household with a mentally ill or depressed parent/caretaker. Contrary to our hypothesis, early maturing women were also more likely to have a history of tonsillitis. These associations were not explained by women’s age at interview, race/ethnicity, level of education, or language dominance. We did not find evidence supporting cumulative childhood adversities as a risk factor for early age at menarche within this sample.

According to the Adaptive Calibration Model (ACM) [58], an individual’s developing stress system (e.g., hypothalamic-pituitary-adrenal (HPA) axis) is an underlying mechanism linking pre-pubertal stressful experiences to adolescent problems through conditional adaptation of pubertal maturation (via the hypothalamic-pituitary-gonadal (HPG) axis). Per this perspective, one’s stress response system is calibrated in response to early environmental experiences/exposures and ultimately regulates traits and behaviors aimed at optimizing the organism’s ability to meet evolutionary goals (e.g., surviving to reproduce), though responses born of hostile environments may lead to higher rates of physical and mental health problems in adulthood [58,59,60]. Research mainly from animal models suggested that the HPA axis developed in conjunction with the HPG axis and worked in parallel in response to external stimuli. Chronic stress, including childhood adversity, could hyperactivate the HPA axis and impair the negative feedback mechanism resulting in a slower recovery of the stress system and metabolic stress (i.e., increased insulin resistance and adiposity) [61]. Downregulation of the HPA system then results in attenuated cortisol profiles [62,63]. The lower cortisol levels coupled with increased insulin and adiposity may accelerate pubertal development because the stress function of the HPA axis was muted, allowing the pubertal and growth hormones sequence via the HPG axis to commence [64]. Therefore, it is plausible that childhood stressful environments associated with childhood adversities may accelerate the onset of menarche through chronic stress stimuli elevating HPA and HPG-axes. More research in this area is clearly needed to explore the multiple and, perhaps, simultaneous HPA-HPG pathways.

Consistent with this model, empirical research showed that the quality of the family environment such as parental maladjustment or family dysfunction, operationalized variably across studies [59,60,65,66,67,68], significantly influenced some girl’s pubertal experience [69,70,71,72,73]. We were able to replicate the inverse association between parental mental illness/depression and age at menarche reported in previous studies [66,67,68,69,74] and speculated that long-term mental illness of a parent/caregiver in our sample decreased the quality of family relationships and household functioning and possibly the socioeconomic position of the family, hence introducing significant early life stress and accelerating reproductive maturation in the exposed women compared with the unexposed. However, we observed no associations between other specific types of adversity (i.e., household alcohol or drug abuse, domestic violence, physical abuse, or emotional abuse), nor the cumulative number or domains of adversities which deviated from prior literature [25,67,71,75]. Differences in population characteristics, the prevalence of adversities, and unmeasured coping mechanisms and/or protective buffers may explain inconsistencies across studies. Notably, we did not ascertain women’s experiences with sexual abuse or parental separation, which may result in a lower prevalence of childhood adversity in the present study as compared with findings from population-based studies [76]. Given the mixed results, future studies should continue to consider cumulative, domains, and a broad range of individual indicators of childhood adversities within cohorts of multiethnic populations in different geographic settings to elucidate their possible contribution to pubertal timing.

Less evidence was available on childhood infections and age at menarche, and to the best of our knowledge, our study was the first to assess the impact of proxy measures for the timing of EBV infection (i.e., tonsillitis and infectious mononucleosis) on menarche. We used self-reported tonsillitis as a proxy for a primary childhood EBV infection and found that women reporting tonsillitis had an earlier mean age at menarche and were 1.7 times more likely to begin menarche before the age of 12. The results were contrary to our hypothesis that a childhood primary infection with EBV would be associated with later age at menarche. According to the life-history theory, with lower exposure to childhood infections, the body would have more energy to invest in growth in reproduction as opposed to the immune system [1,31]. Thus, females exposed to infections in early life might experience slower growth and development [77,78] or possibly have fewer ovarian follicles [79]. However, a trade-off might not be needed in the case of primarily asymptomatic infections. While EBV was not completely asymptomatic given a delayed infection with EBV is associated with recurrent tonsillitis and infectious mononucleosis [45]; an EBV infection is unlike more severe infections that are associated with a later age at menarche. For example, two studies, one measuring HIV infection [35] and the other *Helicobacter pylori* [80], found associations with later onset of menarche where these two types of infections are predominantly symptomatic. The few studies assessing the effect of different types of infection on age at menarche in humans were inconclusive [35,36,80,81]. One prospective cohort study of 101 Taiwanese girls (enrollment age (SD) 4.6 (3.1)) corroborated our findings showing earlier age at menarche in girls who had an earlier spontaneous HBeAg seroconversion and a greater rate of HBsAg clearance, which represented an inactive HBV carrier state [36]. In another study, EBV infection was not associated with an earlier age at menarche in a contemporary cohort of girls; however, in this prospective analysis, more than 50% of the cohort was right-censored [81]. EBV infections are primarily latent and asymptomatic and have even been shown to protect against secondary pathogenic infections to the advantage of the host [82]. Interestingly, our findings would corroborate the hypothesis that a delayed infection with EBV was associated with an increased risk of breast cancer, as an earlier age at menarche is a risk factor for breast cancer [45]. Nonetheless, these findings should be considered with caution. While tonsillitis was used as a proxy for EBV, EBV is not an exclusive etiological cause of tonsillitis [83,84]. We also could not establish a temporal order between the exposure and outcome.

Our findings should be interpreted within the limitations of the study. This study benefitted from including a collection of data on a range of childhood adversity and infectious exposures in a predominantly Hispanic and foreign-born sample, under-represented in prior studies. The retrospectively reported measures of childhood adversity and early life infections could be subject to misclassification due to the sensitive nature of the items or poor recall of experiences occurring several decades in the past. Any misclassification; however, would likely be independent of reports of age at menarche. Data on menarche were collected through a different questionnaire module, prior to asking the women about childhood experiences, thus, likely resulting in underestimation of associations with childhood experiences and age at menarche. Likewise, we relied on women recalling their age at menarche; however, women’s recall of menarcheal age in the midlife has been shown to be generally reliable even in mid- to late-adult life and was validated in previous studies [85]. Although exploratory, we addressed a novel and unexplored question about childhood infection and pubertal maturation, as measured by age at menarche. Using proxies for the timing of early life EBV exposure, we assessed the effect of exposure to tonsillitis and infectious mononucleosis on age at menarche in a diverse sample of women, which had not been tested before in the previous literature. The evidence for infectious exposure and pubertal timing are inconsistent, but theories of development suggest that childhood infections play a role in pubertal timing. Whether EBV exposure affects the biological pathway for earlier or later age at menarche is still unknown. In this study, we assumed that the timing of exposure to EBV infection would occur earlier for tonsillitis than mono, which may not always hold constant. However, given that we observed different findings for the association between tonsillitis and mono with age at menarche may offer some support of this hypothesis and help validate the use of mono as a proxy for delayed EBV infection. Taken together, available data suggested that childhood infections may be associated with the timing of menarche but the direction of these associations and whether they represent causal associations merit further investigation.

## 5. Conclusions

In this cohort of socially diverse women, largely of immigrant background, we noted that mental illness in the household during childhood and a history of tonsillitis were associated with an earlier age at menarche. Other forms of childhood adversity and a history of infectious mononucleosis showed no evidence of association with female pubertal timing. Our findings added to existing research by demonstrating the importance of considering different sources of early life stress and environmental exposures that link childhood experiences to health outcomes across the lifespan. These findings were also timely as existing clinical trials assessing a prophylactic EBV vaccine [37] to potentially reduce the incidence of infectious mononucleosis, but also a range of malignancies, suggest control of this infection is of high priority. Additional research is needed to understand the mechanisms driving and modifiable factors mediating the relationship between childhood adversities, early life infections, and pubertal timing. These factors may help to inform interventions that may reduce the long-term effect of adversities and infections on individuals’ health and wellness.

## Figures and Tables

**Table 1:** Distribution of sociodemographic characteristics, childhood adversities, and early childhood infection for women by menarche status (early, average/late ^1^; N = 400).

Characteristics	All 400	Early Menarche 124	Average/Late Menarche 276	*p*-Value
Age, M (SD)	51.5 (5.6)	51.5 (5.4)	51.5 (5.7)	0.9770
Race/ethnicity, N (%)				0.2033
Hispanic	294 (73.5)	96 (77.4)	198 (71.7)	
Non-Hispanic White	43 (10.8)	8 (6.5)	35 (12.7)	
Non-Hispanic Black	56 (14.0)	19 (15.3)	37 (13.4)	
Other ^2^	7 (1.8)	1 (0.8)	6 (2.2)	
Education, N (%)				0.0669
Less than high school	74 (18.5)	15 (12.1)	59 (21.4)	
High school graduate	97 (24.3)	35 (28.2)	62 (22.5)	
Some college	100 (25.0)	37 (29.8)	63 (22.8)	
Bachelor’s or higher degree	129 (32.3)	37 (29.8)	92 (33.3)	
Nativity, N (%)				0.1021
U.S.-born	135 (33.8)	49 (39.5)	86 (31.2)	
Foreign-born	265 (66.3)	75 (60.5)	190 (68.8)	
Language dominance, N (%)				0.3066
English	188 (47.0)	63 (50.8)	125 (45.3)	
Spanish	212 (53.0)	61 (49.2)	151 (54.7)	
Menarche (years), M (SD)	12.6 (1.9)	10.4 (0.9)	13.6 (1.4)	<0.0001
Cumulative childhood adversities, N (%)	343 (85.8)	106 (30.9)	237 (69.1)	0.7301
None	176 (51.3)	54 (50.9)	122 (51.5)	
1	56 (16.3)	17 (16.0)	39 (16.5)	
2	47 (13.7)	15 (14.2)	32 (13.5)	
3	30 (8.8)	11 (10.4)	19 (8.0)	
4	16 (4.7)	3 (2.8)	13 (5.5)	
5	11 (3.2)	5 (4.7)	6 (2.5)	
6	7 (2.0)	1 (0.9)	6 (2.5)	
Indicators of childhood adversity, N (%)				
Domain: Childhood maltreatment				
Physical abuse	96 (24.0)	31 (25.0)	65 (23.6)	0.7389
Emotional abuse	118 (29.3)	36 (29.0)	81 (29.4)	0.9634
Domain: Parental maladjustment				
Domestic violence	76 (19.0)	25 (20.2)	51 (18.5)	0.6884
Mental illness in household	57 (14.3)	23 (18.6)	34 (12.3)	0.1242
Household alcohol abuse	82 (20.5)	26 (21.0)	56 (20.3)	0.8914
Household substance abuse	28 (7.0)	9 (7.3)	19 (6.9)	0.8243
Member of household went to prison	23 (5.8)	7 (5.7)	16 (5.8)	0.9731
Infection history, N (%)				0.0283
Tonsillitis only	66 (16.5)	30 (24.2)	36 (13.0)	
Mono only	19 (4.8)	4 (3.2)	15 (5.4)	
Both tonsillitis and mono	19 (4.8)	8 (6.5)	11 (4.0)	
Neither tonsillitis nor mono	248 (62.0)	70 (56.5)	178 (64.5)	

Notes: M = mean, SD = standard deviation. ^1^ Early menarche: <12 years; average/late menarche: ≥12 years. ^2^ Other race/ethnicities included Asian, mixed-race. *p* values are from chi-square tests for categorical variables and t-tests for continuous variables.

**Table 2:** Multivariable associations between indicators of childhood adversities and age at menarche (years).

**Childhood Adversities, Cumulative**		**β**	**95% CI**	**Adjusted Mean Age**
No childhood adversity		ref.		13.3
1 childhood adversity		−0.27	−0.85, 0.32	13.1
2 childhood adversities		−0.37	−1.01, 0.28	13.0
≥3 childhood adversities		−0.33	−0.89, 0.23	13.0
**Childhood Adversities, Individual**		**β**	**95% CI**	**Adjusted Mean Age**
Physical abuse	Yes	−0.29	−0.74, 0.16	12.9
	No	ref.		13.2
Emotional abuse	Yes	−0.17	−0.59, 0.25	13.0
	No	ref.		13.2
Domestic violence	Yes	−0.19	−0.68, 0.30	13.0
	No	ref.		13.2
Mental illness in household	Yes	−0.59	−1.15, −0.02	12.7
	No	ref.		13.2
Household alcohol abuse	Yes	−0.03	−0.50, 0.45	13.1
	No	ref.		13.1
Household substance abuse	Yes	−0.13	−0.87, 0.62	13.0
	No	ref.		13.2
Member of household went to prison	Yes	0.25	−0.58, 1.08	13.4
	No	ref.		13.1

Notes: Models for cumulative childhood adversity indicators included complete responses for each of the seven adversity questions (N = 343). Models for individual childhood adversities included complete responses per indicator (range: N = 373 to 388). All models adjusted for age at interview, race/ethnicity, education, and language dominance.

**Table 3:** Multivariable associations between childhood infections and age at menarche (years) (N = 352).

Childhood Infections, Cumulative	β	95% CI	Adjusted Mean Age
Neither tonsillitis nor mono	ref.		13.2
Tonsillitis, no mono	−0.73	−1.26, −0.20	12.5
Mono, no tonsillitis	0.22	−0.69, 1.16	13.4
Tonsillitis and mono	0.45	−0.44, 1.34	13.6

Notes: All models adjusted for age at interview, race/ethnicity, education, and language dominance.

## Appendix A

**Table A1:** Sample eligibility comparisons.

Characteristics	Excluded (N = 122) ^1^	Included (N = 400) ^1^	*p*-Value
Menarche (years), M (SD)	12.85 (1.85)	12.62 (1.94)	0.2313
Education, N (%)			
Less than HS HS Graduate Some College Bachelor’s degree or higher	32 (26.23) 27 (22.13) 27 (22.13) 36 (29.51)	74 (18.50) 97 (24.25) 100 (25.00) 129 (32.25)	0.325
Race/Ethnicity, N (%)			
Hispanic NH White NH Black NH Other	89 (72.95) 14 (11.48) 13 (10.66) 6 (4.92)	294 (73.50) 43 (10.75) 56 (14.00) 7 (1.75)	0.203
Nativity, N (%)			
U.S.-born Foreign-born	35 (28.69) 87 (71.31)	135 (33.75) 265 (66.25)	0.296
Language dominance, N (%)			
English Spanish	52 (42.62) 70 (57.38)	188 (47.00) 212 (53.00)	0.396

Note: ^1^ We excluded data from two women with missing data for age at menarche (one in each group).

**Table A2:** Multivariable associations between childhood adversity domains and mean age at menarche, (N = 343).

Childhood Adversity, Domains	β	95% CI	Adjusted Mean Age
Neither	ref.		13.3
Childhood maltreatment only	0.13	−0.69, 0.72	13.3
Parental maladjustment only	−0.41	−1.05, 0.23	12.9
Both	−0.40	−0.90, 0.11	12.9

Note: All models adjusted for age at interview, race/ethnicity, education, and language dominance.

**Table A3:** Adjusted associations of childhood adversity domains, with early age at menarche (<12 years), relative risk ratios and 95% confidence intervals (N = 343).

Childhood Adversity, Domains	RR	95% CI
Neither	ref.	
Childhood maltreatment only	0.78	0.42, 1.48
Parental maladjustment only	1.07	0.66, 1.74
Both	1.10	0.76, 1.60

Note: All models adjusted for age at interview, race/ethnicity, education, and language dominance.
